# Selective whole-genome sequencing of *Plasmodium* parasites directly from blood samples by nanopore adaptive sampling

**DOI:** 10.1128/mbio.01967-23

**Published:** 2023-12-06

**Authors:** Katlijn De Meulenaere, Wim L. Cuypers, Julia M. Gauglitz, Pieter Guetens, Anna Rosanas-Urgell, Kris Laukens, Bart Cuypers

**Affiliations:** 1 Department of Computer Science, Adrem Data Lab, University of Antwerp, Wilrijk, Belgium; 2 Department of Biomedical Sciences, Malariology Unit, Institute of Tropical Medicine, Antwerp, Belgium; 3 Excellence centre for Microbial Systems Technology, University of Antwerp, Wilrijk, Belgium; The George Washington University Milken Institute of Public Health, Washington, DC, USA

**Keywords:** *Plasmodium falciparum*, nanopore sequencing, adaptive sampling

## Abstract

Whole-genome sequencing of *Plasmodium* is becoming an increasingly important tool for genomic surveillance of malaria. Due to the predominance of human DNA in a patient blood sample, time-consuming laboratory procedures are required to deplete human DNA or enrich *Plasmodium* DNA. Here, we investigated the potential of nanopore adaptive sampling to enrich *Plasmodium falciparum* reads while sequencing unenriched patient blood samples. To compare adaptive sampling versus regular sequencing on a MinION device, a dilution series consisting of 0%–84% *P*. *falciparum* DNA in human DNA was sequenced. Half of the flow cell channels were run in adaptive sampling mode, enriching for the *P. falciparum* reference genome, resulting in a three- to five-fold enrichment of *P. falciparum* bases in samples containing 0.1%–8.4% *P. falciparum* DNA. This finding was confirmed by sequencing three *P. falciparum* patient blood samples with common levels of parasitemia, that is, 0.1%, 0.2%, and 0.6% in adaptive mode. Their estimated enrichment was 5.8, 3.9, and 2.7, respectively, which was sufficient to cover at least 97% of the *P. falciparum* reference genome at a median depth of 5 (lowest parasitemia) or 355 (highest parasitemia). In all, 38 drug resistance loci were compared to Sanger sequencing results, showing high concordance, which suggests that the obtained sequencing data are of sufficient quality to address common clinical research questions for patients with parasitemias of 0.1% and higher. Overall, our results indicate that adaptive nanopore sequencing has the potential to replace more time-consuming *Plasmodium* enrichment protocols in the future.

**IMPORTANCE:**

Malaria is caused by parasites of the genus *Plasmodium*, and reached a global disease burden of 247 million cases in 2021. To study drug resistance mutations and parasite population dynamics, whole-genome sequencing of patient blood samples is commonly performed. However, the predominance of human DNA in these samples imposes the need for time-consuming laboratory procedures to enrich *Plasmodium* DNA. We used the Oxford Nanopore Technologies’ adaptive sampling feature to circumvent this problem and enrich *Plasmodium* reads directly during the sequencing run. We demonstrate that adaptive nanopore sequencing efficiently enriches *Plasmodium* reads, which simplifies and shortens the timeline from blood collection to parasite sequencing. In addition, we show that the obtained data can be used for monitoring genetic markers, or to generate nearly complete genomes. Finally, owing to its inherent mobility, this technology can be easily applied on-site in endemic areas where patients would benefit the most from genomic surveillance.

## INTRODUCTION

The vast majority of human malaria cases are caused by the *Plasmodium falciparum* parasite. As the burden of this disease persists (1), a better understanding of *P. falciparum* population dynamics, and genomic surveillance of drug resistance and diagnostic markers is required (2, 3). The most comprehensive method to simultaneously characterize resistance determinants and study population dynamics is whole-genome sequencing (WGS).

It remains challenging to obtain sufficient amounts of parasite DNA straight from patient blood samples to fulfill WGS requirements, and multi-step wet-laboratory procedures are required (4–10). A blood sample of a patient infected with *Plasmodium* consists of infected red blood cells (RBCs) containing malaria DNA, non-infected RBCs that contain no DNA (no nucleus), and white blood cells (WBCs) that contain human DNA. A low fraction of the red blood cells are infected, typically <2.5% for patients with uncomplicated *P. falciparum* malaria (11). Therefore, the majority of the obtained DNA will be human. To avoid expensive futile sequencing of human DNA, leukocyte depletion and selective whole genome amplification of parasite DNA (sWGA) are commonly used (4, 8–10). Leukocyte depletion techniques should be applied to freshly collected blood samples, which limits the applicability of this technique in low-resource or remote settings where laboratory facilities are not often close to the site of collection (9, 10). sWGA can be performed on frozen blood samples or dried blood spots, but is more expensive and time-consuming compared to leukocyte depletion, and potentially introduces amplification bias (6, 8). The Oxford Nanopore Technologies (ONT) adaptive sampling feature offers a new way to deplete human DNA present in a *Plasmodium* patient blood sample. During nanopore sequencing, DNA is translocated via a protein pore, through which concurrently an ionic current flows. The DNA molecule disturbs this current, generating an electrical signature that corresponds to a set of nucleotides and their modifications (12). Because the voltage across the pore can be reversed, molecules can also be ejected. Signals originating from the DNA molecules that are being sequenced can be classified in real time and used to decide whether a molecule should be ejected or not. This procedure is referred to as “adaptive sampling” and allows for the enrichment or depletion of a target of interest while sequencing (11–14). In addition, ONT devices allow for mobile and real-time sequencing (8, 9), enabling the generation of sequencing data in endemic settings where patients could directly benefit from genomic surveillance. Throughout this manuscript, we refer to nanopore sequencing using an ONT device in adaptive sampling mode as “adaptive nanopore sequencing.”

**Fig 1:**
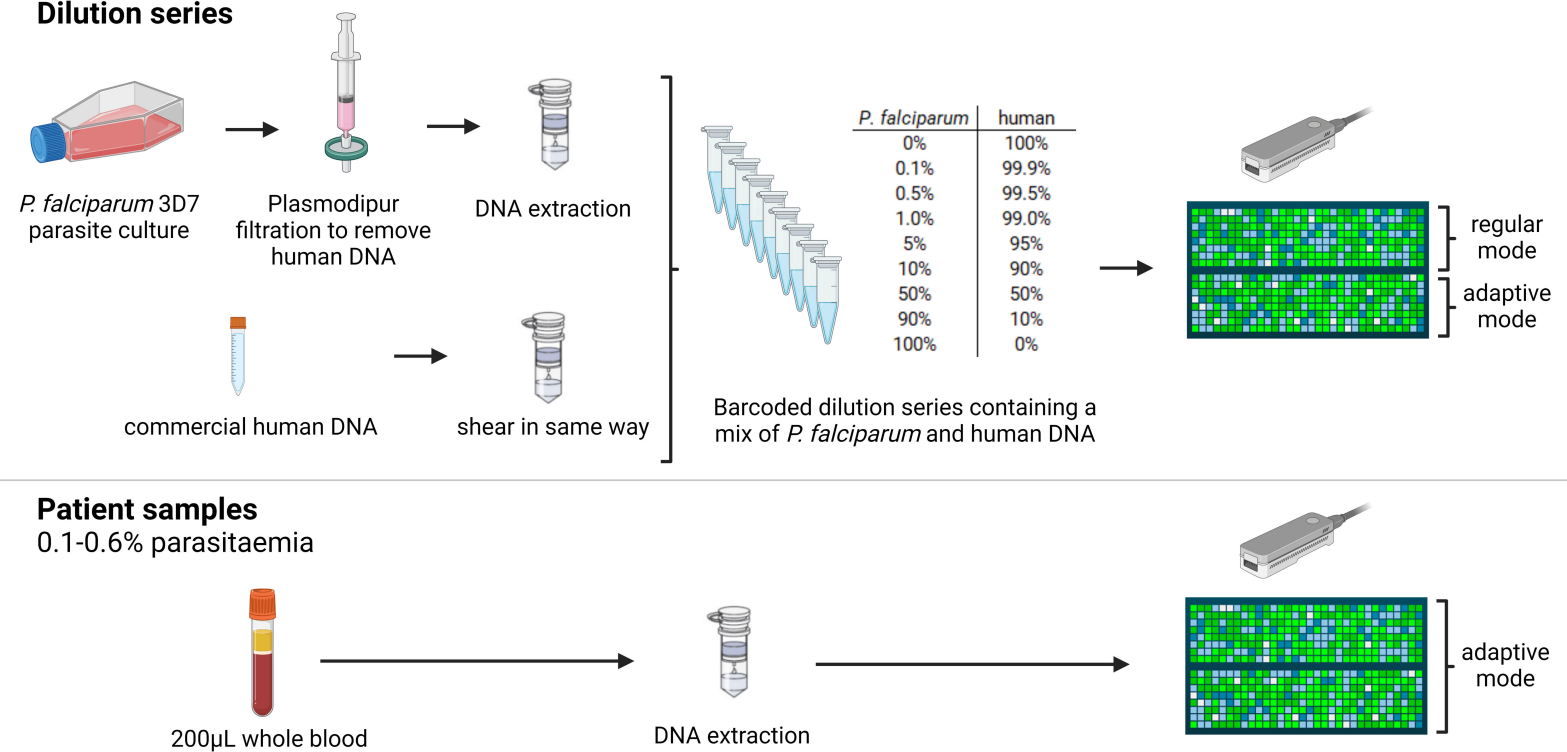
Overview of the experimental setup. First, a dilution series of human DNA mixed with *P. falciparum* DNA was sequenced with half of the ONT flow cell operating in adaptive mode and the other half in regular mode. Next, three *P. falciparum* patient samples were sequenced in adaptive mode, without prior leukocyte depletion or parasite enrichment. Figure created using BioRender.com.

In this paper, we provide a proof-of-concept of the adaptive nanopore sequencing paradigm applied to the characterization of malaria field samples. In particular, we assessed the utility of adaptive sampling for the enrichment of *Plasmodium* DNA in blood samples from human *P. falciparum* patients, without a prior leukocyte depletion or parasite enrichment step. Two experiments were performed (Fig. 1). In the first experiment, we compared adaptive sampling to regular sequencing for a dilution series of human DNA mixed with *Plasmodium* DNA. In the second experiment, three patient samples were sequenced using the adaptive sampling feature. Subsequently, we assessed the quality of the obtained data for genome assembly and the identification of mutations associated with drug resistance or the performance of diagnostic tests. Our results demonstrate that adaptive sampling enables a sufficiently deep sequencing of patient samples with commonly occurring parasitemia levels (0.1%–0.6%). This breakthrough opens up new possibilities for on-site real-time genomic surveillance. It encompasses opportunities for the characterization of strain diversity and detecting drug resistance markers, which has great potential to inform patient treatment.

## RESULTS

### Adaptive sampling enriches sequenced *P. falciparum* bases three- to fivefold in samples with 0.1%–8.4% *P. falciparum* DNA

To test whether and to which degree adaptive sampling can enrich for reads originating from low-abundance *P. falciparum* DNA in a human blood sample, we made a series of nine *P. falciparum* DNA samples diluted in commercial human DNA at different percentages, ranging from 0% *P*. *falciparum* and 100% human DNA to 100% *P*. *falciparum* and 0% human DNA (Fig. 1). Enrichment by adaptive sampling depends on the fragment size of the input DNA (11), which was determined using a Fragment Analyzer for the 0% and 100% *P*. *falciparum* samples. The average fragment size of the DNA from the human sample was circa 39 kb, while the *P. falciparum* DNA peaked at around 46 kb (fragment size distributions in Fig. S1 at https://doi.org/10.6084/m9.figshare.21750476). Samples were pooled and sequenced on one flow cell with half of the channels operating in adaptive sampling mode (enriching for the *P. falciparum* reference genome) and half of the channels operating in regular sequencing mode (Fig. 1).

**Fig 2:**
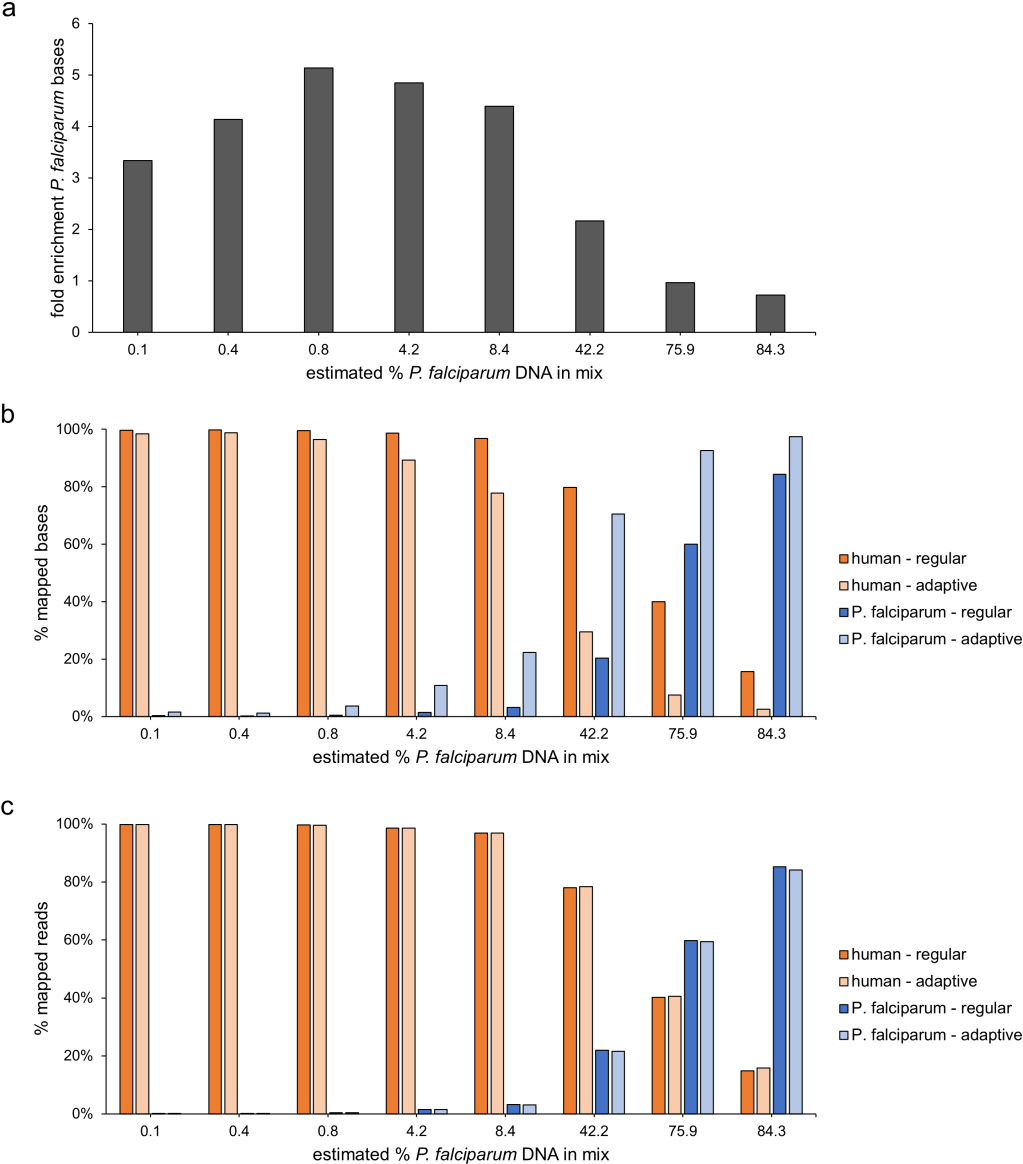
Comparison of human and *P. falciparum* reads, bases, and enrichment for regular and adaptive sampling of the dilution series, consisting of eight samples ranging from 0.1% *P*. *falciparum* and 99.9% human DNA to 84.3% *P*. *falciparum* and 15.7% human DNA. (**A**) Enrichment for *P. falciparum* DNA in adaptive sampling mode. Bars indicate the enrichment for bases mapping to the *P. falciparum* reference genome, taking into account the lower yield in adaptive mode. (**B**) Adaptive sampling increased the number of mapped on-target (*P. falciparum*) bases and decreased the number of off-target (human) bases. The sum of human and *P. falciparum* % of reads equals 100% per sequencing mode. (**C**) Percentages of reads per species remained the same in adaptive sampling mode and regular sequencing mode. The sum of human and *P. falciparum* % of reads equals 100% per sequencing mode.

Adaptive sampling enriched the number of bases mapping to the *P. falciparum* reference genome 3.2-fold on average (range: 0.7- to 5.1-fold) for the different concentrations (Fig. 2A and B). Lower overall yield was previously observed during sequencing runs in adaptive sampling mode, compared to regular mode nanopore sequencing (11, 14). Hence, we calculated the enrichment by dividing the fraction of *P. falciparum* bases sequenced in adaptive mode by the fraction of *P. falciparum* bases in regular mode while normalizing for the lower output in adaptive mode compared to regular mode (1.6× lower for this run).

Further inspection of the read lengths and read fractions per species provided mechanistic insight into the adaptive sampling procedure. The percentage of reads per species remained unaltered (Fig. 2C), and human reads were ejected after sequencing approximately 400 bases, which allowed at least three times more reads to be sequenced (Fig. S2; Table S1 at https://doi.org/10.6084/m9.figshare.21750476). In adaptive sampling mode, the median read length was 391 bp for human reads and 1,701 bp for *P. falciparum* reads (Fig. S2 at https://doi.org/10.6084/m9.figshare.21750476).

Although a dilution series ranging from 0% to 100% of *P. falciparum* DNA was intended, the actual amount of *P. falciparum* DNA present in each of the samples was lower due to incomplete human DNA removal by Plasmodipur filtration in the original *P. falciparum* DNA sample that was used to make the mixed samples (15.7% of reads were human). Samples with low percentages of *P. falciparum* DNA (0.1%, 0.4%, 0.8%, 4.2%, and 8.4%) showed the highest enrichment of 3.3- to 5.1-fold (Fig. 2A). In comparison, the fold enrichment was half or less in samples where *P. falciparum* DNA was more abundant (42.2%, 75.9%, and 84.3%) (Fig. 2A). Due to the higher sequencing efficiency in regular mode, the sample with the highest amount of *P. falciparum* DNA had a fold enrichment of 0.7, showing that the output was higher in the regular sequencing mode. Adaptive sampling substantially increases the amount of sequenced *P. falciparum* bases when sequencing low parasitemia samples, but loses efficiency around 40% *P*. *falciparum* DNA, and highly enriched samples with >75% of *P. falciparum* DNA can be more efficiently sequenced in the regular sequencing mode.

A positive control sample consisting exclusively of commercial human DNA and 0% *P*. *falciparum* DNA was added to estimate the degree of barcode contamination. Of all reads in the positive control sample, 0.17% mapped to the *P. falciparum* reference genome, corresponding to 0.61% of the total amount of bases sequenced for this sample. Homology between these reads and the human reference genome was rare as determined using blastn. Out of 33 and 114 reads that were identified as *P. falciparum* in regular- and adaptive sampling mode respectively, only five had secondary hits to the human reference genome (Table S2 at https://doi.org/10.6084/m9.figshare.21750476). This indicates that the presence of *P. falciparum* reads in the human sample likely resulted from barcode contamination. Conversely, our experiment did not allow an assessment of potential barcode contamination with human reads in the 100% *P*. *falciparum* sample because the Plasmodipur filter removal of human white blood cells is not 100% efficient. Indeed, 15.7% of the reads in the sample consisting of 100% purified *P. falciparum* DNA were identified as human.

### Direct adaptive sequencing of patient blood samples enables comprehensive clinical investigations

Three patient samples were each sequenced on one MinION flow cell (Fig. 1) in adaptive sampling mode to enrich *P. falciparum*, as described above. Competitive read mapping to the human and *P. falciparum* genome revealed that the majority of the reads were human in the three patient samples, and only 0.2% up to 9.7% of the reads were *P. falciparum*. By contrast, the amount of sequenced *P. falciparum* bases varied from 1.7% up to 42.5% in the three patient samples (Table 1). We found that adaptive sampling successfully enriched the amount of sequenced parasite DNA 3.9-fold for patient sample 1, and 5.8-fold for patient sample 2, and 2.7-fold for patient sample 3 (Table 1).

**TABLE 1:** Overview of sequencing coverage, depth, percentage of sequenced *P. falciparum* reads and bases, and the estimated fold enrichment obtained

	Patient sample 1	Patient sample 2	Patient sample 3
Parasitemia	0.23%	0.09%	0.60%
Coverage of the *P. falciparum* 3D7 genome	99.8%	97.4%	99.9%
Median sequencing depth	20	5	355
% of *P. falciparum* bases	6.7%	1.7%	42.5%
% of *P. falciparum* reads	1.1%	0.2%	9.7%
Estimated fold enrichment	3.9×	5.8×	2.7×

For patient samples with commonly occurring parasitemias (~0.1%–0.6%), sufficient coverage and sequencing depth can be obtained for the entire *P. falciparum* 3D7 reference genome with adaptive sampling. Depending on the parasitemia, a genome coverage of 93.4% up to 99.9% was achieved, and a median sequencing depth of 5 up to 355 (Tables S3 to S5 at https://doi.org/10.6084/m9.figshare.21750476). Sequencing depth was uniform across all chromosomes. The mitochondrial genome, however, had a higher median depth of 522, 125, and 6074 for patient samples 1, 2, and 3, respectively, due to its higher copy number (Tables S3 to S5 at https://doi.org/10.6084/m9.figshare.21750476).

Several downstream analyses were conducted to assess whether the data were of sufficient quality to address common clinical and/or research questions. *De novo* genome assembly of *P. falciparum* reads showed that fragmented up to near-complete assemblies could be obtained. For patient sample 1, the assembly size was slightly larger than the current 3D7 genome size (105%), and 138 contigs were obtained, while the assembly size of patient sample 2 was 95% of the reference genome size and 198 contigs were obtained. The high sequencing depth of patient sample 3 resulted in a highly contiguous reference genome with only a few gaps and unassigned contigs and a genome size close to that of 3D7 (99%) (Table S6 at https://doi.org/10.6084/m9.figshare.21750476). Although the *de novo* assemblies of patient samples 1 and 2 were fragmented, many coding genes could be detected, 5,433 (97.7%) and 4,828 (86.8%) for patient samples 1 and 2, respectively. For patient sample 3, 5,066 (91.1%) coding genes were detected.

To assess whether direct sequencing of patient samples with adaptive sampling yields data of sufficient quality for SNP calling [e.g., to screen for single-nucleotide polymorphism (SNPs) implicated in drug resistance or inferring relatedness to other *Plasmodium* strains], we screened the sequencing data for 57 drug resistance-associated markers (15) and compared the results to Sanger sequencing data for 38 of these markers (Data S1 at https://doi.org/10.6084/m9.figshare.21750479). Overall, there was a high concordance of 97% for patient sample 1 and 100% for patient samples 2 and 3 between the results obtained through nanopore sequencing and Sanger sequencing (Table 2). For patient sample 1, we found six resistance-associated SNPs out of the 57 markers that were all sequenced confidently (depth >10). Three of them were heterozygous, which can be attributed to the high multiplicity of infection found in this patient sample (multiplicity of infection [MOI] = 4). This may have resulted in the two mismatches between the nanopore and Sanger sequencing results as well at the *pfcrt* K76T and the *pfmdr1* Y184F loci. For patient sample 2 (MOI = 1), four drug resistance SNPs were found (Table 2). Despite a lower sequencing depth (Table S4 at https://doi.org/10.6084/m9.figshare.21750476), Sanger sequencing confirmed that these variants were correctly called. However, for samples with a similarly low parasitemia with higher MOI, the accuracy of the called variants may decrease. For patient sample 3, 12 resistance-associated SNPs were identified, of which six were heterozygous due to the polyclonality of the infection (MOI = 2). Resistance-associated variants called for patient sample 3 were the same for adaptive sampling mode sequencing, or sWGA combined with regular mode sequencing.

**TABLE 2:** Concordance and discordance between nanopore (adaptive mode) and Sanger sequencing results of 57 WHO drug resistance markers (15) in three *P. falciparum* patient samples^*a*^

	Nanopore result	Sanger result	Drug resistance markers		
			Patient sample 1	Patient sample 2	Patient sample 3
Concordant	Sensitive	Sensitive	33	35	32
	Resistant	Resistant	3	3	3
	Sensitive/resistant	Sensitive/resistant	0	0	3
Discordant	Sensitive	Resistant	1	0	0
	Resistant	Sensitive	0	0	0
	Sensitive/resistant	Resistant	1	0	0
	Sensitive/Resistant	Sensitive	0	0	0
	Resistant	Sensitive/resistant	0	0	0
	Sensitive	Sensitive/resistant	0	0	0
Incomplete data	Sensitive	NA	17	18	13
	Resistant	NA	0	1	2
	Sensitive/resistant	NA	2	0	4

^*a*^ “Sensitive” or “resistant” results are homozygous, while “sensitive/resistant” indicates a heterozygous SNP due to a multiplicity of infection higher than 1 (patient sample 1: MOI = 4, patient sample 2: MOI = 1, patient sample 3: MOI = 2).

Because long nanopore reads are well suited to resolve structural variants, two rapid diagnostic test (RDT) target genes (*pfhrp2*, *pfhrp3*) known to contain large deletions were investigated (2). *Pfhrp3* contained consistent deletions with clear boundaries in the third exon for all three patient samples. A vcf file containing all variants of the three patient samples is provided in Data S2 (at https://doi.org/10.6084/m9.figshare.23710065).

### Adaptive sequencing is fast, flexible, and free of amplification bias

**Fig 3:**
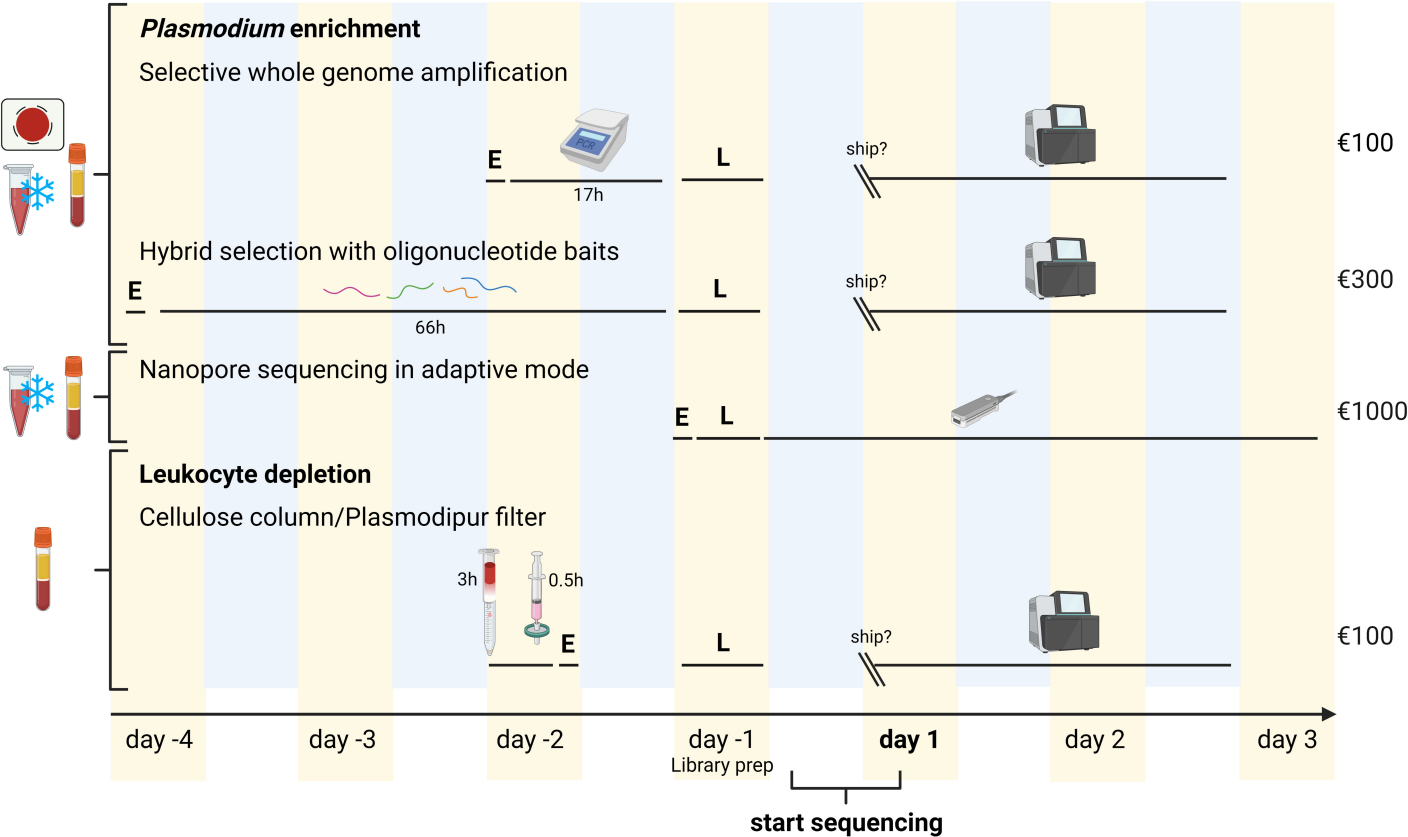
Overview of commonly used methods to increase the amount of *Plasmodium* reads versus human reads, by enrichment of *Plasmodium* DNA or leukocyte depletion, and comparison of duration and cost with adaptive nanopore sequencing. For leukocyte depletion methods, only a fresh whole blood sample can be used, while the other indicated methods can also be carried out on frozen blood or a dried blood spot (indicated on the left). “E” stands for DNA extraction. Prior to library preparation (“L”), sWGA requires a 17-hour amplification step (involving the use of a thermocycler as indicated in the figure), while hybrid selection requires a 66-hour oligonucleotide hybridization step. Leukocyte depletion is faster and requires up to 3 hours using a cellulose column, or 30 minutes with a Plasmodipur filter. On the right, an estimate of the full cost per sample (method-specific preparations, library preparation, and sequencing) at the moment of writing is given. Yellow and blue background colors indicate day and night, respectively. Figure created using BioRender.com.

Compared to other enrichment methods, laboratory costs for adaptive nanopore sequencing are higher. The cost of sequencing one unenriched blood sample on a MinION flow cell was approximately 1,000 euros in July 2023. In comparison, common methods used to sequence a complete *P. falciparum* genome are much more cost-efficient (e.g., 100 euros), based on our *in-house* costs for sWGA and leukocyte depletion and the publication of Melnikov et al. (5) on hybrid selection (5) (Fig. 3).

**Fig 4:**
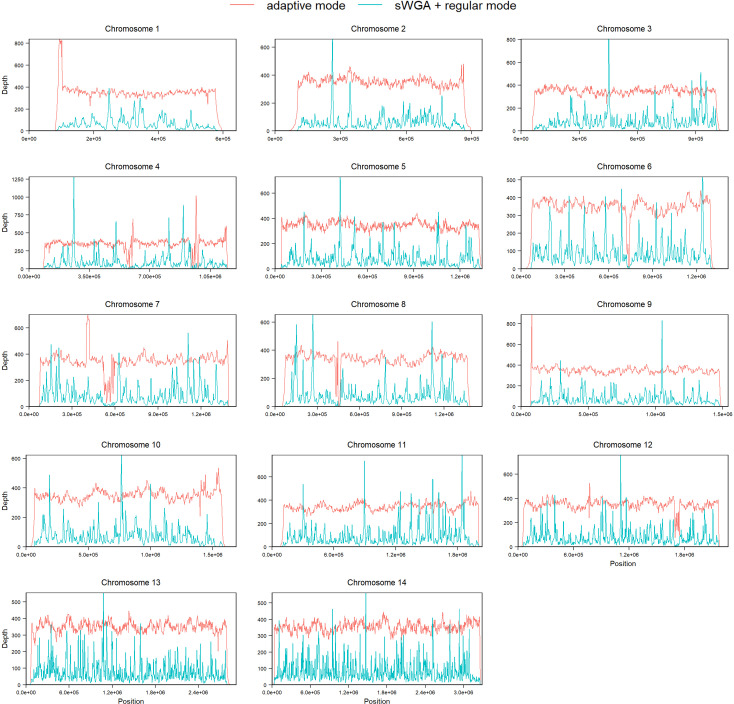
Sequencing depth profiles per chromosome for patient sample 3. *P. falciparum* DNA was enriched using either nanopore adaptive sampling (red) or sWGA (blue) followed by nanopore sequencing in regular mode. Nanopore adaptive sampling shows a more consistent depth profile. Different overall differences in sequencing depth are attributable to technical variation (overloading the sWGA library and differences between flow cells). Subtelomeric regions were removed from the plot (21).

Adaptive nanopore sequencing, on the other hand, offers increased flexibility in terms of time and logistical considerations. The time required to process a sample and obtain *P. falciparum* sequencing data is up to 4 days using nanopore sequencing combined with adaptive sampling, which is shorter compared to the commonly used methods for *P. falciparum* DNA enrichment and sequencing (Fig. 3). This enables rapid data generation, and the shorter sample preparation procedure allows saving costs in terms of labor expenditure. Starting from extracted DNA, a library can be prepared and loaded on a MinION flow cell within 2 hours. Sequencing takes up to 3 days to reach the highest depth, but since the bulk of the data is generated during the first day, variants could be called earlier, and rapid identification of the infecting *Plasmodium* species could already be achieved within a couple of hours. Nanopore basecalling can be done during the run but is preferably carried out afterward (16), which takes up to 1–1.5 additional days. In contrast to leukocyte depletion methods (3, 7, 17), the adaptive sequencing workflow can also start from frozen blood samples, which improves field applicability. The same applies to the other parasite enrichment methods shown in Fig. 3 (sWGA, hybrid selection) (4, 5, 18, 19), but those require a lengthier sample preparation of 2 up to 4 days in comparison to the nanopore sequencing procedure used in this study, which can be done straight from whole blood in a single day. Another advantage of adaptive nanopore sequencing is directly linked to the quality of the generated data. sWGA likely introduces amplification bias and limits the fragment sizes that can be obtained (Fig. S3 at https://doi.org/10.6084/m9.figshare.21750476). We investigated potential amplification bias in sWGA data by performing sWGA and subsequent nanopore sequencing in regular sequencing mode on DNA obtained from patient sample 3. We then searched for markers of amplification bias focusing on two patterns, namely the consistency of read mapping and the presence of chimeric reads, which we compared to the adaptive sampling run of patient sample 3 DNA without prior sWGA enrichment (Fig. 4, Tables 3 and 4). Enrichment by nanopore adaptive sampling showed less peaks in the sequencing depth profiles per chromosome (Fig. 4). This variation in depth is likely a consequence of amplification bias. A second indication of amplification bias was observed as a higher presence of chimeric reads in the sWGA sample (Table 3), which is in line with previous observations (20). Although sWGA results in a larger number of *P. falciparum* reads than adaptive sampling (Table 4), adaptive sampling has the advantage of being more time-efficient and can be used with a PCR-free library prep protocol as we demonstrated here.

**TABLE 3:** Percentage of chimeric reads detected for the three sequenced *P. falciparum* patient samples nanopore sequenced in adaptive mode, compared to patient sample 3 which was selective whole genome amplified (sWGA) followed by Nanopore sequencing in regular mode

Patient sample	Enrichment method	% chimeric *P. falciparum* reads
Patient 1	Adaptive sampling	8.6
Patient 2	Adaptive sampling	9.4
Patient 3	Adaptive sampling	11.9
Patient 3	sWGA	53.3

**TABLE 4:** Percentage of *P. falciparum* and human bases detected for patient sample 3 after either enriching in *P. falciparum* reads using nanopore adaptive sampling, or sWGA followed by Nanopore sequencing in regular sequencing mode

Enrichment method	% *P. falciparum* bases	% human bases
Adaptive sampling	42.5	57.5
sWGA	89.5	10.5

## DISCUSSION

Malaria is among the most deadly infectious diseases in low- and middle-income countries (22), making efficient genomic methods to rapidly analyze *Plasmodium* parasites from patient blood samples a necessity. Nanopore sequencing is a promising method for on-site sequencing of malaria patient samples due to its portability and ease of use. However, its use is not yet widespread, and only one study has reported the use of nanopore technology to whole-genome sequence *P. falciparum* pre-enriched field samples (9). Here, we investigated the feasibility of using adaptive sampling to enrich *Plasmodium* reads while sequencing unenriched samples on a MinION device. We showed that a three- to five-fold enrichment can be achieved for benchmarking samples with 0.1%–8.4% of *P. falciparum* DNA, whereas regular sequencing outperformed adaptive sampling for benchmarking samples with over 75% *P*. *falciparum* DNA. By sequencing three clinical blood samples originating from human patients showing parasitemia levels between 0.09% and 0.60%, we validated the observed enrichment levels (3.9-, 5.8-, and 2.7-fold) which were consistent with the enrichment observed for the benchmarking samples diluted with 0.1%–8.4% of *P. falciparum* DNA. Overall, the degree of enrichment for all samples was similar to previous reports where adaptive sampling enriched target DNA two- up to six-fold in various sample types (11, 23–25).

Sequencing of patient samples in adaptive sampling mode resulted in enough reads to cover at least 97% of the *P. falciparum* reference genome at a mean depth of five up to 355, depending on the parasitic load of the sample. This method can enable rapid insights into important clinical and research questions. For example, we observed a high concordance between drug resistance SNP calls obtained via nanopore and those obtained with Sanger sequencing. This demonstrates that for a monoclonal patient sample, a median sequencing depth of 5 is already sufficient to confidently call SNPs with adaptive nanopore sequencing. In addition, we show the feasibility of producing *de novo* assemblies with near-chromosome level completeness through adaptive nanopore sequencing applied directly to blood samples, provided that the sample parasitemia is on the higher end (>0.6% in our study) and the flow cell output is adequate. This opens up the potential to use this technique for the study of novel gene acquisitions, deletions, duplications, and large structural variants in *Plasmodium*. Particularly in the case of the variable *Plasmodium* subtelomeres (26) or when working with the genetically more diverse *P. vivax* parasite, the efficient generation of *de novo* assemblies through adaptive nanopore sequencing can lead to novel insights.

In comparison to existing WGS methods relying on parasite enrichment (such as sWGA, hybrid selection) (4, 5, 18, 19), the adaptive nanopore sequencing method presented here offers several advantages. Notably, it delivers sequencing results sooner after obtaining the blood sample and increases sample preparation and processing flexibility. The portability of the MinION device allows on-site sequencing in endemic areas, promoting country ownership of data and autonomy of local research groups. However, sWGA, hybrid selection, or leukocyte depletion methods result in higher parasite enrichment than observed for adaptive nanopore sequencing in this study (3–5), and are more cost-efficient at the moment of writing. Despite the advantages of sWGA in enrichment, we showed that sWGA introduces amplification bias, resulting in less consistent depth of coverage profiles and an increase in the number of chimeric reads. Leukocyte depletion methods (cellulose columns or Plasmodipur filter) avoid this issue but add 30 minutes to 3 hours to the total sample processing time, and require fresh blood samples (3, 7, 17). Therefore, leukocyte depletion is only applicable when a laboratory facility is near the sampling site and possesses the capability to process samples on arrival.

In terms of nanopore sequencing cost, we anticipate that prices will decrease over time. In this study, we used one flow cell per patient sample. However, multiplexing can be considered when taking into account MOI, parasitemia, and the variability in output attributed to estimations of DNA quantity for loading and intrinsic flow cell variability. Notably, for patient sample 3, characterized by ≥0.6% parasitemia and MOI = 2, the median sequencing depth of 355 shows the feasibility of multiplexing samples. In addition, the reported data in this study were generated with ONT’s Kit 14 chemistry, which tripled the output of *P. falciparum* bases for the same price compared to the previous Kit 12 chemistry. It should also be considered that the MinION sequencing device itself is cheap compared to the cost of purchasing other sequencing platforms or repeatedly shipping and outsourcing to a sequencing facility, decreasing the barrier to entry.

The nanopore adaptive sampling workflow is less complicated than other existing methods, compatible with frozen blood, and therefore more suited for mobile genomic surveillance in low-resource settings. In addition, several optimizations could be investigated to further improve applicability. The first set of optimization strategies to consider is associated with sample processing and DNA extraction. Up to 500 µL patient blood could be collected by finger prick in an ethylenediaminetetraacetic acid (EDTA) microtainer, as opposed to intravenous puncture as carried out in this study. The ratio of parasite to human DNA could be improved by spinning down the fresh blood sample upon arrival in the laboratory, to remove plasma and buffy coat, as opposed to the frozen whole blood sample that was used here. In addition, DNA extraction protocols could be optimized to obtain longer fragments or deplete short fragments, since longer DNA fragments can lead to higher enrichment via adaptive sampling (11).

Further optimization can be pursued on the level of the adaptive sampling procedure. Algorithmic improvements may further improve the adaptive sampling efficiency by deciding to eject a read faster and/or more accurate. In addition, the adaptive sampling rules can be tailored to match the research question. For analysis of drug resistance markers or genomic surveillance, a predefined set of regions that contain drug resistance SNPs or population genetic markers of interest could be enriched.

A final avenue for optimization involves using the method presented here as a species-agnostic diagnostic tool. Genomic co-characterization of pathogens infecting a patient could improve pathogen surveillance and inform patient treatment. Clinically distinguishing between severe malaria and bacteremia or other febrile illnesses is difficult, especially in low-resource settings (27, 28). Malaria also occurs as a co-infection with human African trypanosomiasis which requires a different treatment regimen (29). Further research is required to investigate whether adaptive sampling-enhanced nanopore sequencing directly from human blood can detect low-abundance DNA, stemming from bacterial or viral infections.

In conclusion, we demonstrated that adaptive-sampling-enhanced nanopore sequencing is a promising method to sequence *P. falciparum* directly from blood samples. It shortens the time to retrieve a *P. falciparum* genome from a human blood sample compared to existing methods, can be deployed in most clinical settings, and generally provides enough sequencing depth to address common research questions, including calling drug resistance loci and generating informative *de novo* assemblies. With future improvements in mind, both in sample collection and sequencing technology, adaptive nanopore sequencing holds promise to become a field-deployable tool for rapid *Plasmodium* sequencing.

## MATERIALS AND METHODS

### Samples and DNA extraction

A dilution series of simulated patient samples containing an estimated amount of 0%, 0.1%, 0.5%, 1%, 5%, 10%, 50%, 90%, and 100% *P*. *falciparum* DNA was made by mixing *P. falciparum* 3D7 DNA with commercially available human DNA (Promega, G304A) to a total amount of 400 ng DNA. Actual amounts were lower due to incomplete removal of human DNA in the *P. falciparum* sample used to make the mixed samples and are estimated to be 0%, 0.1%, 0.4%, 0.8%, 4.2%, 8.4%, 42.2%, 75.9%, and 84.3%. *P. falciparum* strain 3D7 was *in vitro* cultured to 5%–8% parasitemia, after which the parasites were harvested, and the remaining human white blood cells were depleted with Plasmodipur filtration (EuroProxima) as described by Auburn et al. (3). DNA was extracted from 400 µL RBCs using the QIAamp DNA Mini kit (Qiagen). The commercial human DNA was processed using the same QIAamp DNA Mini columns to obtain similar DNA fragment sizes for both the human and *P. falciparum* DNA. Fragment sizes were assessed with the Fragment Analyser (Agilent, kit DNF-464-33—HS Large Fragment 50 Kb) (Fig. S1 at https://doi.org/10.6084/m9.figshare.21750476).

Clinical samples originated from three patients who presented themselves at the Institute of Tropical Medicine Antwerp (ITM) travel clinic with a *P. falciparum* infection. DNA was extracted from 200 µL whole blood (stored in EDTA) using the QIAamp DNA Mini kit (Qiagen). Parasite density (parasites/µL) was determined by varATS qPCR, based on a protocol adapted from Hofmann et al. (30). Sample 1 contained 11,687 parasites/µL, which corresponds to a parasitemia of approximately 0.23% (infected RBCs/RBCs). Sample 2 contained 4,508 parasites/µL, which corresponds to approximately 0.09% parasitemia. Sample 3 contained 30,200 parasites/µL, which corresponds to approximately 0.60% parasitemia.

### Library preparation and sequencing

The human and *Plasmodium* DNA dilution series consisted of nine mixed samples that were barcoded and prepared for sequencing using the Native Barcoding Kit 24 (Q20+) (SQK-NBD112.24) following the manufacturer’s protocol (version NBE_9134_v112_revE_01Dec2021). The resulting barcoded library was loaded on one R10.4 flow cell, and sequencing was performed on a MinION sequencer with MinKnow v23.03.6 (ONT). Channels 1–256 were operating in adaptive sampling mode, with the 3D7 reference genome (PlasmoDB v56) (31) listed as the target genome for enrichment. Channels 257–512 were operating in regular sequencing mode. After 72 hours, 4.26 gigabases (Gb) of data were generated and the run was stopped.

Three additional libraries were prepared for adaptive nanopore sequencing from clinical samples 1, 2, and 3 using the Ligation Sequencing Kit V14 (SQK-LSK114) according to the manufacturer’s protocol. Sequencing was conducted on a MinION sequencer (R10.4.1 flow cell) coupled to MinKnow (ONT), at a sequencing speed of 400 bps. Table S7 (at https://doi.org/10.6084/m9.figshare.21750476) details the quantity of input DNA, timing of wash steps (Flow Cell Wash Kit EXP-WSH004) and reloading of the second part of the library, and the total sequencing time for each sample. Three libraries originating from unenriched DNA were sequenced in adaptive sampling mode, enriching for the 3D7 reference genome.

In addition, 30 µL (408 ng) of patient sample 3 was sWGA as previously described (4) followed by library preparation and sequencing as described above. Unlike the previous sequencing runs, this library underwent sequencing in regular sequencing mode as it was pre-enriched using sWGA. We observed a lower output during this sequencing run compared to the previous runs, likely due to the shorter fragment size of the sWGA enriched DNA which may have resulted in overloading of the flow cell.

Patient samples 1 and 2 were also sequenced before with the SQK-LSK112 kit, but because kit SQK-LSK114 clearly delivered higher output and therefore better *P. falciparum* sequencing depths (comparison in Table S8 at https://doi.org/10.6084/m9.figshare.21750476), only SQK-LSK114 data will be shown and discussed.

### Basecalling and species identification

Basecalling was performed offline using Guppy GPU v6.0.7 or higher (see Table S7 at https://doi.org/10.6084/m9.figshare.21750476 for the exact versions per sample). All data were basecalled using the super-accurate model (version in Table S7 at https://doi.org/10.6084/m9.figshare.21750476). Quality metrics were assessed using pycoQC v2.5.2 (https://github.com/a-slide/pycoQC) and Nanoplot v1.28.1 (32). For the dilution series experiment, reads were separated according to sequencing mode (adaptive sampling versus regular sequencing) using seqtk v1.3 (https://github.com/lh3/seqtk).

Reads were competitively mapped using minimap2 v2.17-r941 (33) to a reference fasta file consisting of the human (GRCh38) and *P. falciparum* 3D7 (PlasmoDB v58) reference genomes, to minimize false-positive mapping. Unmapped (bitwise flag 4) and alternatively mapping (bitwise flag 256) reads were removed with samtools v1.10 (34) from coordinate sorted bam files, after which *P. falciparum* and human reads could be split based on their genome-specific coordinates. The number of mapped bases was extracted from the samtools stats report [“bases mapped (cigar)”], and the number of mapped reads and supplementary (chimeric) reads from the samtools flagstat report. Depth per position was obtained with the samtools depth command and further analyzed in R (35). Coverage was calculated as the fraction of the reference genome that was covered by at least one uniquely mapping read. Depth was calculated as the average number of reads that cover a position in the reference genome.

Reads of the dilution series’ positive control sample (100% human DNA) that mapped to the *P. falciparum* reference genome were subjected to an additional analysis using blastn v2.13.0+ (36, 37) to confirm whether these reads originated from *P. falciparum* or showed homology to the human reference genome. Reads were first converted to the FASTA format using seqtk and compared to a custom database consisting of *Homo sapiens* genome assembly GRCh38.p14 and *P. falciparum* GCA_000002765.3 (retrieved on 03/08/2022). For reads with hits to the human reference genome, a maximum of five hits with the highest bitscore were extracted and further inspected.

Taxonomic read classification was performed using Kraken2 (--confidence 0.1) (15) combined with the PlusPF database (retrieved from https://benlangmead.github.io/aws-indexes/k2 on 10/05/2022) to assess the species composition of the patient samples and check for potential contaminants. Most reads originated from either *Homo sapiens* or *P. falciparum*. A small fraction of identified reads originated from *Toxoplasma gondii* (Table S9 at https://doi.org/10.6084/m9.figshare.21750476). To account for the possibility of a database search artifact, we utilized the aforementioned blastn methodology to compare each read to a custom database consisting of *Homo sapiens* genome assembly GRCh38.p14, *P. falciparum* GCA_000002765.3 and *Toxoplasma gondii* GCF_000006565.2_TGA4 (retrieved on 17/07/2023). This revealed that the aforementioned reads were predominantly derived from the human reference genome and not from *T. gondii*.

### Qualitative comparison of sequencing output between *P. falciparum* patient samples

To estimate the extent to which adaptive sampling enriched *P. falciparum* DNA in the clinical samples, we compared the fraction of sequenced *P. falciparum* bases to the sequenced fraction of *P. falciparum* reads. The latter is an approximation of the unknown fraction of *P. falciparum* DNA that is present in the patient samples. To take into account that adaptive sampling decreases the total yield compared to regular mode sequencing (11), this number is divided by 1.6 (yield correction factor determined *via* the dilution series experiment) to get the *P. falciparum* enrichment in comparison to a run in regular mode.

Variants of the clinical samples were called with Clair3 v1.0.1 (model r941_prom_sup_g5014, diploid mode) (38). To validate the called variants, a set of genes with known drug resistance markers (*pfdhfr*, *pfmdr1*, *pfcrt*, *pfK13*, and *pfcytb*) was PCR amplified and Sanger sequenced (Genewiz, Germany) according to existing methods (39–42). MOI, that is, a polyclonal infection, was determined by MSP1 and MSP2 genotyping (43). Gvcf variant files of the three patient samples were merged into a single vcf file using GATK (44) CombineGVCFs and GenotypeGVCFs (v4.1.4.1) and were made available in Data S2 (at https://doi.org/10.6084/m9.figshare.23710065).

*De novo* genome assembly of *P. falciparum* reads originating from patient samples 1, 2, and 3 was carried out using Flye v2.9 (45) with the option --nano-hq and the --genome-size parameter set to 23 m. One round of polishing based on the *P. falciparum* mapped reads was carried out with Medaka v1.6.1 (medaka_consensus, model r1041_e82_400bps_sup_variant_g615) (https://github.com/nanoporetech/medaka). Scaffolding of contigs against the *P. falciparum* 3D7 reference (PlasmoDB v58) was performed using RagTag v2.1.0 (46), without prior correction to maintain true biological structural variation. The resulting scaffolds were annotated with Companion v1.0.2 (47).

## Data Availability

The data for this study have been deposited in the European Nucleotide Archive (ENA) at EMBL-EBI under accession number PRJEB57715. FASTQ files originating from patient samples were purged from human reads for ethical reasons.
